# Major Bioactive Alkaloids and Biological Activities of *Tabernaemontana* Species (Apocynaceae)

**DOI:** 10.3390/plants10020313

**Published:** 2021-02-05

**Authors:** Clarissa Marcelle Naidoo, Yougasphree Naidoo, Yaser Hassan Dewir, Hosakatte Niranjana Murthy, Salah El-Hendawy, Nasser Al-Suhaibani

**Affiliations:** 1 School of Life Sciences, Westville Campus, University of KwaZulu-Natal, Private Bag X54001, Durban 4000, South Africa; naidooclarissa5@gmail.com (C.M.N.); naidooy1@ukzn.ac.za (Y.N.); 2 Plant Production Department, College of Food and Agriculture Sciences, King Saud University, P.O. Box 2460, Riyadh 11451, Saudi Arabia; mosalah@ksu.edu.sa (S.E.-H.); nsuhaib@ksu.edu.sa (N.A.-S.); 3 Department of Horticulture, Faculty of Agriculture, Kafrelsheikh University, Kafr El-Sheikh 33516, Egypt; 4 Department of Botany, Karnatak University, Dharwad 580003, India; hnmurthy60@gmail.com; 5 Department of Agronomy, Faculty of Agriculture, Suez Canal University, Ismailia 41522, Egypt

**Keywords:** alkaloids, Apocynaceae, biological activity, pharmacological properties, *Tabernaemontana*

## Abstract

Several species belonging to the genus *Tabernaemontana* have been well researched and utilized for their wide-ranging biological activities. A few of the most prominent species include *Tabernaemontana divaricata*, *Tabernaemontana catharinensis*, *Tabernaemontana crassa*, and *Tabernaemontana elegans*. These species and many others within the genus often display pharmacological importance, which is habitually related to their chemical constituents. The secondary metabolites within the genus have demonstrated huge medicinal potential for the treatment of infections, pain, injuries, and various diseases. Regardless of the indispensable reports and properties displayed by *Tabernaemontana* spp., there remains a wide variety of plants that are yet to be considered or examined. Thus, an additional inclusive study on species within this genus is essential. The current review aimed to extensively analyze, collate, and describe an updated report of the current literature related to the major alkaloidal components and biological activities of species within the genus *Tabernaemontana*.

## 1. Introduction

The genus *Tabernaemontana* belonging to the family Apocynaceae was named by a German physician and botanist, J. Th. Muller [1]. At present, approximately 100 species belonging to this genus have been distributed in tropical and subtropical regions around the world, including Africa, Asia, Oceania, and the Americas [2]. *Tabernaemontana* species consists of flowering shrubs and small-medium-sized trees, which habitually grow in the savannahs, rocky outcrops, and forest understories [3]. Characteristic features of the genus include tubular white flowers, follicular fruit with seeds embedded within a yellow to reddish aril, and a milky or watery latex exudate, which is often found in wounded species [4]. Due to the latex content, plants within this genus are usually called “milkweed” and are often used for their biological activities [2,5,6]. Plants within the genus *Tabernaemontana* obtain a profusely high alkaloid content, usually displaying pharmacological activity [2]. Furthermore, monoterpene indole and bisindole alkaloids are the major classes of alkaloids within the genus, and other compounds include terpenes, lactones, steroids, phenolics, and flavonoids [1]. Over 67 species have been investigated for indole alkaloids, of which 470 isolations of approximately 240 structurally different bases have been detected [2,3,7].

A few of the most intensively studied *Tabernaemontana* species include *T. divaricata*, also known as “Crape Jasmine”, which occurs in the tropical regions of southern China, India, and Thailand [8]. Crape Jasmine is intensively utilized as an aphrodisiac, tonic, and a purgative [8]. According to Van Beek et al. [1], in western India, latex is used for inflammation and wound healing. *T. catharinensis*, usually recognized as “Snakeskin”, is a small tree native to Brazil and is found in many surrounding countries [9]. This species is used frequently in traditional medicine for the removal of warts and as an antidote for snakebites [10]. *Tabernaemontana crassa*, also known as “Adam’s apple flower”, occurs in Africa, DR Congo, and north of Angola [11]. A substantial number of alkaloids have been identified in *T. crassa*, possibly suggesting its medicinal properties [1]. This species has several traditional uses: a local anesthetic; and in the treatment of malaria, wounds, sores, and abscesses [1,12]. *Tabernaemontana corymbosa*, locally, referred to as “Jelutong badak”, is a wild shrub or small tree that primarily occurs in Indonesia, Laos, Thailand, Vietnam, Malaysia, and China [13]. All parts of *T. corymbosa* are regularly used in ethnobotany [13,14,15]. In South Africa, the genus is represented by two species: *Tabernaemontana ventricosa* and *T. elegans* [16,17]. *Tabernaemontana ventricosa*, frequently termed the “Forest toad tree”, is a small-medium-sized tree that is native to Africa [18]. All parts of the tree contain a white milky latex substance [18]. Due to other better-known *Tabernaemontana* species, little or no research has been carried out on *T. ventricosa*. However, despite the lack of research, *T. ventricosa* remains ethnomedicinally used in South Africa to promote wound healing, aid in pain relief, treat high blood pressure, reduce fever, and contains antiamoebic activity [18,19]. *Tabernaemontana elegans*, commonly called the “Toad tree”, is a small tree found in river fringes and coastal scrub forests [20]. In Zimbabwe and Mozambique, the fruit is regularly eaten when ripe, as it is deemed to contain medicinal properties [1]. The coagulated latex is used as a preventative measure for bleeding (Styptic), root decoctions are ingested for pulmonary disease and chest pains, and seeds and stem bark are used to treat heart disease and cancer [1,21,22].

Currently, the massive potential of countless *Tabernaemontana* species has led to the investigation of various plant crude extracts, fractionations, chemical constituents, and isolated compounds [2]. These novel assessments are exceedingly valuable as it may be applied for the detection of innovative compounds that could be used to improve the pharmacological background of this genus [2,7]. However, despite the consistent usage of *Tabernaemontana* species in traditional medicine systems and the confirmation of their biological activities, various species are yet to be investigated for their chemical properties and biological activities [2,3]. The review will explicitly elaborate on species within *Tabernaemontana*, with emphasis on their biological activities that have been recently published.

## 2. Major Bioactive Components and Biological Activities of *Tabernaemontana* Species

The *Tabernaemontana* genus acquires a generous source of monoterpene indole alkaloids, which are derived from the aromatic acid tryptophan and the iridoid terpene secologanin [6]. Monoterpene indole alkaloids have been found to exhibit numerous skeletal types, namely seco-tabersonine alkaloids, bis-vobtusine-type alkaloids, and bis-vosbsinyl-ibogan indole alkaloids [3,11]. Currently, there are > 1800 structurally diverse monoterpene-derived indole alkaloids that have been classified within *Tabernaemontana* [3,6]. Another major class of alkaloids identified in this genus is heterodimeric bis-indole alkaloids [6]. This compound has been characterized by the biosynthesis of dimeric structures from two independent-class alkaloids [6]. A summary of the major alkaloids isolated from species within the genus *Tabernaemontana* is displayed in Table 1.

For centuries, several *Tabernaemontana* species, such as *T. divaricata*, *T. catharinensis*, *T. crassa*, and *T. elegans*, have been exploited in traditional and folk medicine for the treatment of illnesses and the prevention of diseases and ailments, such as sore throat, hypertension, abdominal pain, and pulmonary disease [9,20,70,71,72]. A variety of chemical compounds extracted from many parts of *Tabernaemontana* species reportedly contain alkaloids, which exhibit biological activities, such as antimicrobial, antioxidant, anti-inflammatory, anticholinesterase, antineurodegenerative, anticancer, antidiabetic, antivenom, larvicidal, antihypertensive, wound healing, analgesic, and many other activities [2,3,6,9,71,72]. The details of the isolated compounds and respective pharmacological properties of a few *Tabernaemontana* species are summarized in the following paragraphs.

The findings of Nicola et al. [9] provide scientific support to the frequently used traditional medicinal plant *T. catharinensis*. The outcomes of the study revealed the presence of major alkaloids, such as 16-epi-affinine, coronaridine-hydroxyindolenine, voachalotine, voacristine-hydroxyindolenine, 12-methoxy-n-methyl-voachalotine, and a derivative of voacristine or voacangine (Table 1). It was suggested by Nicola et al. [9] that these chemical constituents exhibited anticholinesterase activity and can be recommended for the future treatment of neurodegenerative disease. According to Mairura [45], a substantial amount of indole alkaloids have been identified and isolated from the stem bark, rootbark, and seeds of *T. crassa*. Major alkaloids include those of the ibogan class, such as coronaridine, mono- and di-methoxy derivatives of isovoacangine, conopharyngine, and the aspidospermatan-class apparicine (Table 1). Mairura [45] explained that the plant is highly toxic, as crude ethanolic extracts were found to be lethal to test subjects. Conversely, the study of Kuete et al. [73] investigated the toxicity of hydro-ethanol stem-bark extracts and the results showed no toxicological activity, thus suggesting a novel source of naturally produced drugs. Ingkaninan et al. [53] investigated the phytochemical properties of the flowers, leaves, stems, and root extracts of *T. divaricata*. Additionally, four isolated compounds, namely 19,20-dihydrotabernamine, 19,20-dihydro-ervahanine A, conodurine, and tabernaelegantine A, were screened for biological activity. The findings revealed that the extracts and respective compounds displayed high antiacetylcholinesterase activity. Furthermore, studies have shown that isolated compounds from *T. divaricata*, such as conophylline, were effective against in several cell lines [74]. Previous phytochemical research has shown *T. elegans* to contain several monoterpenoid indole alkaloids of which 24 were previously isolated [56]. The major indole alkaloidal components extracted from the whole plant and root bark of *T. elegans* were vobasine, dregamine, and tabernaemontaninol [6,16,55]. A recent study by Pallant et al. [20] reported the isolation and identification of alkaloids in the root extract of *T. elegans*. Major components observed were dregamine and voacangine, which exhibited significant antibacterial activity against Gram-positive bacteria and *Mycobacterium* species.

Despite the variety of biologically active compounds displayed in the above-mentioned species and Table 1, several other *Tabernaemontana* species, such as *T. ventricosa*, lack an in-depth chemical and pharmacological investigation. A few studies have observed the bioactivity of *T. ventricosa* [1,75]. Van Beek et al. [1] reported that the alkaloid akuammicine, belonging to the strychnan class, exhibited opioid activity in opiate receptor studies. The same group investigated the antibacterial, antifungal, and antimalarial activities of *T. ventricosa* extracts; however, no activity was observed in vitro. Mehrbod et al. [75] investigated the effect of *T. ventricosa* plant extracts on the influenza A virus. This investigation supported the study of Van Beek et al. [1], as the results concluded that the leaf extracts of *T. ventricosa* were ineffective against the influenza A virus. Due to the traditional uses of *T. ventricosa* being very similar to those of other well-known studied species, little to no studies have been conducted on *T. ventricosa*, thus it is necessary to evaluate the complete medicinal potential of this species and other *Tabernaemontana* species to determine its probable pharmacological activities. Considering the several uses of *Tabernaemontana* species in traditional medicine, many of their proposed biological activities have been confirmed, others invalidated, while countless species remain undefined [2]. Additionally, the improvements in science and medicine have allowed the discovery of new properties of extracts, fractionations, and the identification and isolation of novel compounds [2].

### 2.1. Antioxidant Activity

Antioxidants are identified as molecules or compounds that regulate the process of autoxidation either by intersecting the movement of free radicals or directly constraining their formation [76,77]. Medicinal plants are often recognized for their rich source of antioxidants, which include phenolic acids, phenolic diterpenes, flavonoids, volatile oils, carotenoids, and anthocyanidins [77]. These compounds target free radicals by quenching oxygen molecules, breaking antioxidant chains, donating hydrogen molecules, or acting as reducing agents [76,78]. Therefore, antioxidants are suggested to decrease oxidative stress, improve immune function, and increase healthy longevity [76,77,78]. Several factors can alter the antioxidant capacity of a certain species; these include the rate of reaction between the samples and the reactive species and the concentration ratio between the antioxidant and the target [2]. There are multiple methods used to determine the antioxidant activity of plant species; however, various methods may result in variation of the results [79]. Many species within the *Tabernaemontana* genus have been investigated for their antioxidant activity using different techniques, which include the inhibition and scavenging activity of reactive oxygen species and reactive nitrogen species, reducing capacity, and metal-chelating capacity [2]. The most frequently studied species within the genus is *T. catharinensis* [9]. Table 2 summarizes the antioxidant properties of *Tabernaemontana* species. Boligon et al. [80] investigated the crude leaf extracts and fractions of *T. catharinensis* by using the thiobarbituric acid reactive substances technique. Ethyl acetate and n-butanol fractions, yielding a half-maximal inhibitory concentration (IC_50_) of 6.71 ± 0.19 μg/mL and 26.15 ± 0.08 μg/mL, respectively, displayed optimal results. Furthermore, the same study also assessed the 1,1-Diphenyl-2-picrylhydrazyl (DPPH) inhibition of *T. catharinensis* extracts, which exhibited good results, with an IC_50_ value of 4.64 ± 1.25 to 27.78 ± 0.93 mg/mL [80]. Additionally, Nicola et al. [9] examined the antioxidant activity of the alkaloidal fraction in the branch and leaf ethanolic extracts of *T. catharinensis*. The findings of the study revealed significant antioxidant activity from the alkaloidal fraction, with an IC_50_ of 37.18 μg/mL [9].

### 2.2. Anti-Inflammatory Activity

Inflammation is defined as a compound biological process that involves an adamant response of an organism to injury or damage of tissue [109]. The development of inflammation is often induced by microbial infection, chemical injury, cell injury, and death [110]. The consequences of these inducers are primarily indicated by pain, redness, heat, and swelling, which arise due to the deviations in blood flow, capillary permeability, and afferent nerve fibers [111,112]. Subsequently, these changes imitate the restoration of inflamed tissue and constrain additional damage to the organism [109]. Based on the characteristics of inflammation, this composite process is divided into two major types known as acute and chronic inflammation [72,109,113]. Acute inflammation occurs almost instantly, or a few hours following injury, and usually displays symptoms of redness, heat, and edema [72]. Whereas, chronic inflammation occurs over an extended interval and is histologically characterized by the occurrence of lymphocytes and macrophages, which subsequently results in the development of fibrosis and necrosis tissue [114]. The standardized protocol for the evaluation of anti-inflammatory activity comprises ex vivo and in vivo experiments [2]. Many *Tabernaemontana* species have been assessed for anti-inflammatory activity. Table 3 summarizes those anti-inflammatory properties of *Tabernaemontana* species. Jolly et al. [93], investigated the anti-inflammatory activity of ethanolic flower extract obtained from *T. divaricata*. Mice models were subjected to acute carrageenan and chronic formalin [93]. The results showed significant anti-inflammatory activity in both models at a dose of 100 mg kg^−1^, in comparison to the standard reference drug diclofenac (25 mg kg^−1^) [38]. Furthermore, Jain et al. [115] examined the in vivo anti-inflammatory activity of *T. divaricata* leaves. In this study, hexane fractionations containing a profuse source of flavonoids were tested on male albino mice [115]. The results revealed extensive anti-inflammatory activity, which displayed enhanced results in comparison to the positive drug indomethacin [115].

### 2.3. Antimicrobial Activity

Antimicrobials are defined as complex compounds that constrain the development of microorganisms at diminutive concentrations [128]. These compounds are often described as secondary metabolites and are regularly produced and extracted from medicinal plants or microorganisms [129]. The efficiency of antimicrobial activity is dependent upon several factors, such as various microbial strains, technique (in vivo or in vitro assay), and type of sample [2]. Studies have reported the evaluation of *Tabernaemontana* extracts as natural antibiotics [3]. Monoterpenoid indole alkaloids, such as voacamine type and 3-hydroxy-iboga, are biologically active compounds and are reportedly used as antimicrobial agents, inhibiting the growth of bacteria, fungi, and parasites [3]. Table 4, Table 5, Table 6 and Table 7 summarize the antifungal, antiviral, antibacterial, and antiamoebic properties of *Tabernaemontana* species.

#### 2.3.1. Antifungal Activity

Singh et al. [54] investigated the antifungal activity of a biologically active compound from *T. divaricata*. A major compound from the *Tabernaemontana* genus, coronaridine (Table 1), was identified and isolated from the ethanolic extract of *T. divaricata* [54]. In the study, coronaridine displayed weak antifungal activity against *Penicillium chrysogenum*, comparable with nystatin [minimum inhibitory concentration (MIC) 9.8–14.0 mg/mL] [54]. Conversely, Boligon et al. [132] investigated the dichloromethane and n-butanol fractions of ethanolic leaf extracts from *T. catharinensis*. The dichloromethane fraction showed significant activity against the fungal strains *C. albicans*, *C. glabrata*, *C. neoformans*, *S. cerevisiae*, *A. flavus*, and *A. fumigatus*, with MIC ranging from 31.25 to 1000 mg/mL [132].

#### 2.3.2. Antiviral Activity

Boligon et al. [132] evaluated the dichloromethane, ethyl acetate, and n-butanol fractions from the ethanolic extracts of *T. catharinensis*. The extracts and fractions displayed substantial antiviral activity on herpes simplex virus type 1 (HSV-1) in contrast to acyclovir (IC_50_ 1.5 mg/mL) [132]. Furthermore, significant antiviral activity was observed from the dichloromethane and ethyl acetate fractions. The dichloromethane and ethyl acetate fractions of the bark-stem fractions showed IC_50_ values of 2.62 and 2.88 µg/mL, respectively, whereas the fractions of the leaves showed IC_50_ values of 0.6 and 2.21 µg/mL, respectively [132]. It is suggested that biologically active compounds, such as steroids, terpenoids, and phenolics, found in the dichloromethane, ethyl acetate, and n-butanol fractions are accountable for the antiviral activity [2,132].

#### 2.3.3. Antibacterial Activity

Over the past 50 years, extensive research has been conducted about the advancements in antibacterial medicines [175]. Moreover, due to the reoccurrence of multidrug-resistant bacteria, there is an excessive necessity for the development of novel and innovative antibacterial agents against those displaying multidrug resistance [175,176]. Approximately 25% of modern medication has been established on the core basis of plant-related compounds [177]. Thus, the active screening of medicinal plants is imperative for the discovery of new antibacterial compounds [178]. The indole alkaloids of *Tabernaemontana* exhibit a wide range of pharmacological activities, including antibacterial activity against Gram-positive and Gram-negative bacteria [133]. Gindri et al. [150] investigated the antibacterial activity of ethanolic extract and its fractions from the leaves of *T. catharinensis*. The extracts and fractions were tested against multiple bacterial strains, such as *S. aureus*, *Aeromonas* sp., *Micrococcus* sp., *P. mirabilis*, *E. coli*, *K. pneumoniae*, *E. faecalis*, and *P. aeruginosa*, which were comparable against the antibiotics ampicillin (MIC = 8.0 mg/mL), cefoperazone (MIC = 16.0 mg/mL), and imipenem (MIC = 0.06 mg/mL). The findings showed positive results against *Micrococcus* sp., *P. mirabilis*, and *P. aeruginosa* (MIC values of 31.3, 62.5, and 62.5 mg/mL, respectively) [150].

#### 2.3.4. Antiamoebic Activity

Parasitic infections are regarded as one of the leading contributors to human health problems and are often distributed via contaminated food and water sources [179]. Amoebiasis is a lethal disease that arises from the ingestion of pathogenic microorganisms and occurs predominantly in tropical areas, including China, Mexico, the Eastern portion of South America, South-East and West Africa, Asia, and the Indian subcontinent [180,181]. The protozoan parasite *E. histolytica* is dominant in these regions and affects approximately 12% of the world’s population, whilst being responsible for copious mortality rates, ranging from 40,000 to 110,000 per year [180,182]. Since there is no vaccine against *E. histolytica*, metronidazole (MNZ) is regularly used to treat infection against amoebiasis; however, there are serious consequences of the drug, such as amoebic resistance and several side effects, including impaired physical and mental development [183]. Due to the severe side effects of MNZ, many infected people have opted for a more natural approach using traditional medicine [180]. Several *Tabernaemontana* species are often used in various parts of the world for their antimicrobial, antiparasitic, and antiamoebic action. Van Beek et al. [1] investigated a variety of *Tabernaemontana* species to establish their antiamoebic activity. In the study, approximately 15 *Tabernaemontana* species were tested against the protozoan *E. histolytica*. The study exhibited adequate results, as four extracts from three species showed promising activity below 0.5 mg/mL against the parasite protozoan *E. histolytica* [1]. Additionally, Uwumarongie et al. [141] evaluated the antiamoebic activity of *T. pachysiphon* root and stem bark extracts. The findings of their study showed relatively high antiamoebic activity against *E. histolytica* [141].

### 2.4. Anticancer and Cytotoxicity

The irrepressible division of cells habitually leads to the formation of cell masses, which are frequently termed as ‘growths’ or ‘tumors’ [184]. These masses are classified as malignant (cancerous) or benign (noncancerous) and are often influenced by several characteristics, such as an irregular diet, genetic factors, and ecological aspects [185,186]. These negative influences give rise to an amplified rate of cancer, since approximately 18.1 million people are expected to be diagnosed with cancer [187,188,189]. However, with the consistent applications of effective cancer treatments, such as radiotherapy, surgery, immunotherapy, and chemotherapy, these values may subside [190,191]. However, among these treatments, chemotherapy remains challenging due to the occurrence of multidrug resistance (MDR), which is defined as the resistance of tumors to chemotherapeutic agents [192]. Considering the above, the constant discovery of anticancer agents using medicinal plants has displayed minimal side effects and act as modulators of MDR. Thus, recently, several medicinal plants, especially within the genus *Tabernaemontana*, have been screened to evaluate their potential effect on the growth and development of cancerous cells [2].

The study conducted by Pereira et al. [33] investigated the isolation, structure, and chemical constituents present in the root bark of *T. catharinensis*. Twenty-seven compounds were detected in the ethanol and *n*-butanol extracts, however, only 12 compounds were identified. Approximately three compounds, namely, ibogamine, 3-oxo-coronaridine, and 12-methoxy-4-methylvoachalotine (MMV) (Table 1), showed substantial cytotoxicity against SKBR-3 breast adenocarcinoma and C-8161 human melanoma tumor cell lines [33]. Thind et al. [193] examined the cytotoxic properties of *T. divaricata*. In the study, various leaf extracts prepared in a range of solvents (chloroform, methanol, ethyl acetate, and hexane) were tested against the following cell lines: HCT-15 (colon), HT-29 (colon), 502,713 (colon), MCF-7 (breast), and PC-3 (prostate) [193]. Ethyl acetate extract was the most promising as it displayed significant cytotoxic activity against the colon cell line (502713) with a low dose of 10 µg/mL [193]. Additionally, the chloroform extracts also showed considerable cytotoxicity against three colon cell lines with a slightly higher dosage of 30 µg/mL [193].

In another study, Rumzhum et al. [96] screened the leaf extracts of *T. divaricata* using a brine shrimp bioassay. The results of the study revealed possible cytotoxicity (LC_50_ = 1 µg/mL) in comparison to the positive control, vincristine sulphate (LC_50_ = 0.3 µg/mL) [95]. Figueiredo et al. [67] reported the cytotoxicity of *T. salzmannii* root and leaf extracts on human leukemia cells (THP-1). In this study, nine alkaloids were isolated; however, only two alkaloids, isovoacangine and voacangine (Table 1), displayed extensive cancer cell death, with an IC_50_ of 52.11 and 61.40 µM, respectively [67]. The above-isolated compounds from various *Tabernaemontana* species are frequently used to treat a range of cancer types, such as breast, germ and renal, leukemia, lung, testicular, ovarian, and colon cancer [17,194,195,196,197,198].

Recently, Rosales et al. [189] also evaluated the anticancer properties of indole alkaloids *of T. catharinensis*. In the study, six compounds were identified, namely, 16-*epi*affinine, 12-methoxy-*n*-methyl-voachalotine, affinisine, voachalotine, coronaridinehydroxyindoline, and ibogamine (Table 1) [189]. The isolated indole alkaloids were tested in vitro against several cell lines, including tumor cells A375 (melanoma cell line) and A549 (adenocarcinomic human alveolar basal epithelial cells), and non-tumor Vero cells (African green monkey kidney epithelial cells) [189]. In vitro toxicity showed the fractionation containing affinisine demonstrated toxicity against A375, with an IC_50_ of 11.73 μg/mL, and may be a chemotherapeutic agent for A375 melanoma cells [189]. Table 8 summarizes the anticancer properties of *Tabernaemontana* species.

### 2.5. Acetylcholinesterase Activity

Alzheimer’s disease (AD) is defined as an advanced chronic and aggressive neurodegenerative disease, which is habitually accompanied by the severe disturbance of multiple cortical functions, including memory impairment, judgment, orientation, understanding, language learning capacity, and personality changes [235,236,237]. Clinically, this cognitive disorder is often characterized by the occurrence of several amyloidal beta-peptide plaques, neurofibrillary tangles, atrophy of basal forebrain cholinergic neurons, oxidative stress, and reduced neurotransmitter levels [9,238,239]. According to Adewusi and Steenkamp [240], AD is amongst the leading disorders worldwide as it is liable for approximately 50–60% of dementia in elders. Moreover, a dramatic incline, possibly affecting 7–10% of individuals over 65 and 40% of persons over 80 years, is anticipated within the next 50 years, without the intervention of rehabilitation [235,240,241]. Popular methods for the treatment of AD are often based on the cholinergic hypothesis, which suggests that the impairment of memory is directly related to a reduction in the function of cholinergic in the brain [241]. Thus, several approaches regarding the enhancement of acetylcholine levels using acetylcholinesterase (AChE) inhibitors are frequently investigated [239,241,242]. Currently, approved AChE inhibitors include tacrine, donepezil, rivastigmine, and galanthamine [238,241,242]. However, despite the beneficial effects on cognitive functioning, these inhibitors have displayed undesirable side effects, such as gastrointestinal issues, nausea, vomiting, and reduced bioavailability [243,244]. Considering the latter, the discovery of alternative natural AChE inhibitors displaying minimal side effects is essential [238,239,244]. Therapeutic plants have been investigated for their complex compounds that contain natural and innovative AChE inhibitors [241,242]. Several *Tabernaemontana* species have been recognized and investigated for their monoterpene indole alkaloids, which have demonstrated AChE inhibitory activity [245]. Ingkaninan et al. [241] investigated the AChE inhibitory activity of methanolic extracts of *T. divaricata*. In the study, extracts (0.1 mg/mL) were tested in vivo using rats as test models [241]. The results were promising as approximately 90% of AChE activity was observed. Furthermore, most recently, Athipornchai et al. [245] investigated the AChE inhibitory activity of the methanol, n-hexane, and ethyl acetate extracts of *T. pandacaqui* flowers. The results revealed that the ethyl acetate extract displayed the strongest AChE inhibitory activity, with inhibition of 35.4% at 5 mg/mL [245]. Considering the latter, prior and recent studies have shown the potential of plant AChE inhibitors, which should be further investigated. Table 9 summarizes the acetylcholinesterase activities of *Tabernaemontana* species.

### 2.6. Other Activities

Khan [98] investigated the gastrointestinal effects of the methanol extract from *T. divaricata* flowers. In the study, a rat pyloric ligation-induced gastric ulceration model was used to evaluate the potential effects, along with the standard drug omeprazole (8.0 mg/kg) [98]. It was revealed that the extract reduced the amount of gastric juice, free and total acidities, ulcer index, and pH of gastric acid produced [98]. The standard drug, omeprazole showed a percentage protection of 89.8%, and the extract 79.5% [98]. In another report, Khan et al. [250] further examined the methanol extract from the flowers of *T. divaricata*, using a range of concentrations (125.0, 250.0, and 500.0 mg/kg, p.o). Some inducers, such as aspirin and ethanol, were tested against gastric ulcers [209]. The standard positive control was misoprostol [250]. Several parameters, such as catalase, superoxide dismutase, mucin, and total protein, were measured and displayed a reduced index when treated with extracts. It has been suggested that the gastrointestinal effects of the extracts occur through an antioxidant pathway [250].

The antidiabetic activity of *T. divaricata* methanol extract was examined on alloxan-induced diabetic rats [251]. The results displayed substantial antidiabetic activity, with an additional reduction in the effect of oxidative damage observed in rats [251]. The extracts exhibited a similar mechanism in relation to the positive control, glibenclamide. The study of Kanthlal et al. [251] recommends that the methanol extract may alert the insulin receptors, therefore triggering a stimulation or production of beta-stem cells in the pancreas of the test subjects. The compound conophylline, which is often isolated from serval *Tabernaemontana* species, displayed antidiabetic activity [252]. Furthermore, conophylline was efficient in inducing the activity of activin A in AR42J cells, which in turn stimulated modification in endocrine cells [252]. The same compound was tested against diabetic rats [253]. In the study, increased plasma levels in normal and streptozotocin-induced diabetic rats were observed along with a significant decrease in blood glucose levels, indicative of antidiabetic activity [253]. In rat pancreatic acinar carcinoma cells and cultured rat pancreatic tissues, the compound conophylline was found to rapidly produce beta-cell differentiation, thus inducing differentiation into insulin-producing cells [74,253].

*Tabernaemontana catharinensis* is often used for its antivenom properties [2]. Almeida et al. [202] examined the antivenom effects. The alkaloidal aqueous fractionation from the root bark of *T. catharinensis* was tested in vivo using *Crotalus durissus terrificus* [202]. It was observed that the extracts were able to extensively defuse the poisonous action of the venom [202]. Núñez et al. [254], investigated the antivenom activity of *T. elegans*. The study observed potential inhibition against *Crotalus durissus cumanensis* venom [254]. The antivenom potential of *T. alternifolia* root extract was tested in vitro and in vivo against *Echis carinatus* venom [255]. A range of pharmacological assays, including lethal toxicity determination, hemorrhagic, and neutralization studies, were carried out using chick embryo models [255]. The extracts showed promising results, since fibrinogen degradation, hemorrhage, and the venom-induced edema were significantly reduced in the models [255]. Recently, Vineetha and coauthors [116] investigated the in vitro and in vivo inhibitory effects of *T. alternifolia* methanolic root extract against *Naja naja* venom. Similarly, in their previous study, the fibrinogenolytic, direct, and indirect hemolytic activities for the neutralization of the venom were observed [116,255]. The results of the study yet again showed great potential, since fibrinogen and hemolytic were neutralized effectively, and the edema ratio was significantly reduced [116,255]. The latex of several *Tabernaemontana* species is habitually utilized for its wound-healing effects [3]. Subsequently, *T. catharinensis* is known for its many medicinal uses, including wound-healing effects [2].

### 2.7. Silver Nanoparticles (AgNps)

Recent trends in science have promoted the synthesis of AgNps in the field of nanotechnology [256]. Nanoparticles are extremely small materials that exhibit nanoscale dimensions ranging from 1–100 nm [257]. Due to their nanoscale size, these particles display a large surface area to volume ratio [258]. The properties of AgNps, such as their size, shape, and morphology, have enhanced their activity and thus are used in an extensive range of applications, such as health, medicine, food, textiles, and agricultural sectors [257]. Many metals have been evaluated for the synthesis of nanoparticles, such as gold (Au), copper (Cu), and alloy of silver (Ag^+^); however, comparable to these metals, reports have shown that Ag^+^ is not considered a hazardous substance [259]. Gorchev and Ozolins [260] reported that minor amounts of Ag^+^ were absorbed in laboratory test subjects ranging between 0% and 10%. The two main methods used to obtain nanoparticles comprise of a “top-down” approach, which can be described as the process whereby nanoparticles are reduced in size until they reach a suitable material; conversely, the “bottom-down” approach involves the development of nanoparticles from an elemental entity, such as atoms and molecules [261]. The top-down method consists of chemical and physical techniques that are often energy consuming and produce imperfect nanoparticles [262]. Moreover, the bottom-down technique includes biological methods, which regularly produces colloidal dispersions of homogenous particles with fewer defects [263]. Current research has shown that the biological synthesis (i.e., the use of living organisms) of AgNps has driven investigations towards a “greener synthesis” approach, which is simple, cost-effective, environmentally friendly, and easily upscaled for large-scale synthesis [256,264]. According to Chouhan [258], the method of biological synthesis using a greener approach is relatively simple, as it requires less time and energy comparable to physical and chemical methods. This approach involves incubating crude aqueous extracts obtained from various plants or plant organs with an aqueous solution comprised of a metal salt, such as silver nitrate (AgNO_3_) [261]. The reaction between the metabolites in the plant extract then reduces the metal ions in solution, thus metal nanoparticles are produced [261]. The use of environmentally friendly plant extracts was discovered to generate a considerable number of AgNps using AgNO_3_ as an inorganic metal [258]. Reported studies have found that AgNps exhibit significant antimicrobial activity and low toxicity to humans [258,265,266].

Recently, several AgNps investigations have been conducted using *Tabernaemontana* species, with *T. divaricata* being the most investigated species (Table 10). In the study by Devaraj et al. [256], various *T. divaricata* extracts were used for biosynthesis, and characterized, and assessed for cytotoxicity against MCF-7 cell lines. The analysis showed that the average particle size ranged from 22.85 nm and the biosynthesized nanoparticles showed potential cytotoxicity to human breast cancer cells (MCF-7) [256]. It is highly probable that these nanoparticles could be regularly used in various sectors, such as medical, cosmetic, and food applications [256]. In another study conducted by Anbukkarasi et al. [267], *T. divaricata* extracts were used for biosynthesizing nanoparticles and the resulting biosynthesized nanoparticles were tested in vivo to prevent the formation of cataracts in selenite-induced cataractogenesis in Wistar rat pups [267]. It was revealed that rats induced with AgNps treatment displayed a reduction in lenticular alterations compared to plant extract-treated rats [267]. The results of the study recommend that biosynthesized AgNps using *T. divaricata* extracts may provide limitations of selenite-induced cataractogenesis in vivo, while simultaneous sustaining standard lenticular calcium homeostasis by avoiding adjustments in important lenticular proteins [267].

## 3. Conclusions

The current review established an inclusive assessment of the major alkaloidal compounds within species belonging to the genus *Tabernaemontana*, which demonstrated pharmacological potential. The various secondary metabolites derived from *Tabernaemontana* species, such as terpenes, lactones, steroids, phenolics, flavonoids, and alkaloids, are often utilized in ethnobotany for their curative effects. Furthermore, these bioactive components have displayed numerous biological activities, including antimicrobial, antioxidant, anti-inflammatory, anticholinesterase, antineurodegenerative, anticancer, antidiabetic, antivenom, larvicidal, antihypertensive action, wound healing, and analgesic effects. However, despite the presence of biologically active chemical compounds within the genus *Tabernaemontana*, many species lack chemical and biological evaluation. Thus, further research is crucial to gain insight about the bioactive compounds and relative pharmacological activities of this esteemed genus.

## Figures and Tables

**Table 1:** Major alkaloids isolated within the genus *Tabernaemontana.*

Species	Reported Alkaloids	References
*Tabernaemontana angulata*	Voacangine, voronaridine	[23]
*Tabernaemontana catharinensis*	Isovoacangine, coronaridine, heyneanine, 16-epiaffinine, catharinensine, 16-decarbo-methoxyvoacamine, conodurine, ibogamine, tabernanthine, voacangine, 3-hydroxyvoacangine, 3-hydroxycoronaridine, 3-oxocoronaridine, catharanthine, voacangine hydroxyindolenine, rupicoline, coronaridinepseudoindoxyl, tetraphyllicine, olivacine, 6 N-hydroxyolivacine, 2-N-oxyolivacine, Nb-demethylvoacamine, voacamidine, tabersonine, 19-epivoacristine, 3-(2-oxopropyl) coronaridine, 12-methoxy-4-methylvoachalotine, voacristine, coronaridinehydroxyindolenine, voacristinehydroxyindolenine, vobasine, voachalotine, voachalotine	[24,25,26,27,28,29,30,31,32,33,34]
*Tabernaemontana coriaceae*	Taberpsychine, vincadifformine, minovincine, 3-oxominovincine, voacorine, epi-(20)-voacorine	[35]
*Tabernaemontana corymbosa*	Conoliferine, conodiparine A, conodiparine C, tronoharine, voastrictine, vobatricine, conodiparine E, conodirinine A, conodirinine B	[36,37,38,39,40,41]
*Tabernaemontana crassa*	Conoduramine, 19-hydroxyconopharyngine, crassanine, ibogamine, coronaridine, isovoacangine, conopharyngine, apparicine	[11,35,42,43,44,45]
*Tabernaemontana dichotoma*	16,22-dihydro-16-hydroxyapparicine, dichomine, vallesamine, voacamine	[46,47]
*Tabernaemontana divaricata*	16-epi-affinine, coronaridine-hydroxyindolenine, voachalotine, voacristine-hydroxyindolenine, 12-methoxy-n-methyl-voachalotine, conofoline, conophyllidine, 3S-cyanocoronaridine, 5-hydroxy-6-oxocoronaridine, 5-oxocoronaridine, 6-oxocoronaridine, ibogamine, voacangine, 3-ethoxyvoacangine, voaharine, voalenine, coronaridine, heyneanine, voacristine, voacamine, decarbomethoxyvoacamine, 19,20-dihydroervahanine, 19,20-dihydrotabernamine, 19,20-dihydro-ervahanine A, conodurine, tabernaelegantine A	[9,35,36,43,44,48,49,50,51,52,53,54]
*Tabernaemontana elegans*	Apparicine, dregamine, vobasine, dregamine, tabernaemontaninol, voacangine	[20,55,56]
*Tabernaemontana heterophylla*	Voacangine, coronaridine, 19-heyneanine, vobasine, affinisine, olivacine	[57]
*Tabernaemontana hystrix*	Ibogaine, iboxygaine, voacangine, coronaridine, voacristine, 19-epivoacristine, iboxygaine, hydroxyindolenine, montanine, vobasine, olivacine, ibogamine, affinine, hystrixnine, affinisine, Nb-methylaffinisine, coronaridine, 3-oxocoronaridine, 5-oxocoronaridine, ibogamine-5,6-dione, coronaridinehydroxyindolenine, vobasine, 12-methoxy-voachalotine	[58,59,60]
*Tabernaemontana pachysiphon*	Conodurine, 3-hydroxyconopharyngine,16-epiisositsirikine	[1,44]
*Tabernaemontana laeta*	Vobasine, affinine, normacusine B, geissoschizol, voacamine, conodurine, vobasine, affinine, akuammidine, affinisine, geissoschizol, voacamine, conodurine, coronaridine, conoduramine, voacangine, isovoacristine, Nb-methylvoachalotine, tabernamine, ibogamine, conopharyngine, voacangine hydroxyindolenine, voachalotine oxindole, normacusine B, pericyclivine, dehydrovoachalotine, Nb-methylvoachalotine, olivacine	[61,62,63,64,65]
*Tabernaemontana rupicola*	Rupicoline, montanine	[66]
*Tabernemontana salzmannii*	Voacangine, isovoacangine, (3S)-hydroxyisovoacangine, coronaridine, 3-oxo-coronaridine, (19S)-heyneanine, voachalotine, olivacine	[67]
*Tabernaemontana siphilitica*	Isobonafousine	[68]
*Tabernaemontana solanifolia*	Voacangine, isovoacangine, coronaridine, heyneanine, isovoacristine, voacangine hydroxyindolenine, vobasine, voachalotine, 12-methoxy-Nb-methylvoachalotine, voacamine	[69]
*Tabernaemontana stapfiana*	Ibogamine	[35,43,44]

**Table 2:** Antioxidant activities of extracts and compounds isolated from *Tabernaemontana* species.

Species	Part/Exudate	Extract/Compound	Models/Methods	References
*Tabernaemontana alternifolia*	Root and leaf	Methanol, chloroform, dichloromethane, and dichloroethane	2,2-Diphenyl-1-picrylhydrazyl (DPPH) method	[81,82,83,84,85,86]
*Tabernaemontana catharinensis*	Branch, leaf, root, stem, stem bark, and fruit	Supercritical fluid extraction, essential oils, ethanol, alkaloidal fractions, 16-epi-affinine, voacangine, voacristine hydroxy indolenine, ethyl acetate, hydroethanol, and fractions	DPPH method, radical reduction-in vitro, ferric reducing antioxidant power (FRAP), 2,2-azino-bis-(3ethylbenzothiazoline-6-sulphonic acid (ABTS), coupled oxidation of carotene and linoleic acid assay—Male Wistar diabetic rats	[9,10,80,87,88,89,90,91,92]
*Tabernaemontana coronaria*	Flower	Petroleum ether, alcohol, and water	Hydroxyl and superoxide radicals in vitro, nitric oxide (NO) scavenging, and lipidperoxidation inhibition activity	[93,94]
*Tabernaemontana corymbosa*	Root, stem, and leaf	Petroleum ether, chloroform, methanol, and water	DPPH method, metal chelation, and reducing power methods—In vitro	[95]
*Tabernaemontana divaricata*	Leaf, flower, stem, root, and latex	Methanol, aqueous, ethanol, petroleum ether, hexane, chloroform, and ethyl acetate, ethyl-4-n-octyl benzoate, ethyl-4-n-decyl benzoate, and digalactosyldeconate.	FRAP, DPPH method—in vitro, hydrogen peroxide (H_2_O_2_) free radicals, reducing power—in vitro, superoxide anion radical scavenging, NO, ABTS, H_2_O_2_ scavenging, and Aꞵ_25–35_ peptide, Novel object recognition test (NOR), crystal violet staining and lipid peroxidation	[82,83,96,97,98,99,100,101,102,103,104,105]
*Tabernaemontana heyneana*	Leaf, stem, bark, and trunk	Ethanol and sodium hypochlorite	Total phenolic content and DPPH method	[106,107]
*Tabernaemontana recurva*	Whole plant	Methanol	Total phenolic content	[108]

**Table 3:** Anti-inflammatory activities of extracts and compounds isolated from *Tabernaemontana* species.

Species	Part/Exudate	Extract/Compound	Cell Lines/Models/Methods	References
*Tabernaemontana alternifolia*	Root	Methanol	Lethal toxicity annulation murine models	[116]
*Tabernaemontana bufalina*	Aerial parts	Coronaridine and pandine	Reduction of lipopolysaccharide (LPS)—induced NO production	[117]
*Tabernaemontana catharinensis*	Stem bark and leaf,	Ethanol, ethyl acetate, dichloromethane, n-butanol, and hydroethanolic	Carrageenan-induced rat paw edema in Wistar albino male rat models, irritant contact dermatitis models in mice, paw edema parameters in pain models involved with TRPA1 activation and dermatitis models	[118,119,120,121,122,123]
*Tabernaemontana divaricata*	Flower, leaf, stem, and aerial parts	Ethanol, ethyl acetate, aqueous, methanol and hexane fraction	Carrageenan and formalin induced—mice models, reduction of interleukin (IL)-6 secretion and tumor necrosis factor (TNF)-α production, Wistar rat models and reduced croton oil–induced edema in mice models	[93,100,102,115,124]
*Tabernaemontana pachysiphon*	Leaf	Tubotaiwine and apparicine	Mouse abdominal constriction testing	[125]
*Tabernaemontana pandacaqui*	Stem	Ethanol and alkaloid fraction	Carrageenan-induced rat paw edema models	[126]
*Tabernaemontana pauciflora*	Root	Voacangine, coronaridine and 3-(2-oxopropyl)-coronaridine	Mice and Guinea pig models	[127]

**Table 4:** Antifungal activities of extracts and compounds isolated from *Tabernaemontana* species.

Species	Part/Exudate	Extract/Compound	Cell Lines/Models/Methods	References
*Tabernaemontana alternifolia*	Root and leaf	Methanol, chloroform, acetone, ethanol, aqueous, methanol with 9% of water and 1% of acetic acid	*Aspergillus terreus*, *Scopulariopsis* sp., *Aspergillus niger*, *Gibberella fujikuroi*, *P. chrysogenum*, *Candida albicans*, *Rhizopus mucor*, *Aspergillus parasiticus* and *Trichoderma viridians. (T. viride)*	[81,130]
*Tabernaemontana angulata*	Aerial parts	Chloroform and methanol	*C. albicans* (ATCC 10231)	[131]
*Tabernaemontan catharinensis*	Aerial parts, leaf, root bark, root, and bark	Methanol, dichloromethane, n-butanol fractions of ethanol, ethanol, 12 methoxy-Nb-methylvoachalotine	*Microsporum canis*, *Microsporum gypseum*, *Trichophyton mentagrophytes*, *Trichophyton rubrum*, *Epidermophyton floccosum*, *C. albicans*, *Candida glabrata*, *Cryptococcus neoformans*, *Saccharomyces cerevisiae*, *Aspergillus flavus*, *Aspergillus fumigatus* and *T. rubrum* strains	[52,132,133,134]
*Tabernaemontana dichotoma*	Root bark, stem bark, leaf, fruit, and seed	Ethanol and methanol	*A. niger* (ATCC 16904), *C. albicans* (ATCC 10235), *A. niger* (MTCC 281), *C. albicans* (ATCC 10231), *P. chrysogenum* (MTCC 2725), *Phanerochaete chrysosporium* (MTCC 787), and *Ralstonia entropha* (MTCC 1255)	[135,136,137]
*Tabernaemontana divaricata*	Flower and leaf	Ethanol, methanol and aqueous	*P. chrysogenum*, *Malassezia furfur* and Poisoned food technique in vitro	[54,136,138,139]
*Tabernaemontana elegans*	Root	Ethanol	*C. albicans* (ATCC 10231) and *C. albicans* (NHLS 255)	[140]
*Tabernaemontana pachysiphon*	Root bark	Ethanol	*A. niger* and *C. albicans*	[141,142]
*Tabernaemontana solanifolia*	Leaf, bark, and root	Methanol	*C. albicans* B311 strain	[69]
*Tabernaemontana stapfiana*	Leaf, fruit, root, and stem bark	Methanol fractions-hexane, dichloromethane, ethyl acetate and methanol	*C. albicans* (ATCC 90028), *C. neoformans* (ATCC 66031), *M. gypseum* (KMR 101), and *T. mentagrophytes* (KMR 100)	[143]

**Table 5:** Antiviral activities of extracts and compounds isolated from *Tabernaemontana* species.

Species	Part/Exudate	Extract/Compound	Cell Lines/Models/Methods	References
*Tabernaemontana catharinensis*	Stem bark	Dichloromethane, ethyl acetate, and n-butanol fractions of ethanol	Herpes simplex virus type 1 (HSV-1)	[132]
*Tabernaemontana cymosa*	Bark and seed	Ethanol, lupeol acetate and voacangine	Dengue virus strains, DENV-2/NG, and DENV-2/16681, in cultured Vero or U937 cells, and Chikungunya Virus	[144,145]
*Tabernaemontana elegans*	Leaf and stem	Ethanol	HSV-1	[146]
*Tabernaemontana laeta*	Leaf, stem, and latex	Hydroethanol	HSV-1, vaccinia virus Western Reserve (VACV-WR), and encephalomyocarditis virus (EMCV)	[147]
*Tabernaemontana pachysiphon*	Root and stem bark	Ethanol	HSV-1, poliovirus, and Semlicki Forest virus	[148]
*Tabernaemontana ventricosa*	Leaf	Methanol	Influenza A virus (IAV)	[75]

**Table 6:** Antibacterial activities of extracts and compounds isolated from *Tabernaemontana* species.

Species	Part/Exudate	Extract/Compound	Cell Lines/Models/Methods	References
*Tabernaemontana alternifolia*	Stem bark and root	Aqueous, petroleum ether, methanol, chloroform, acetone, ethanol, dichloromethane, dichloroethane, and ethyl acetate	Methicillin-resistant *S. aureus* (MRSA), vancomycin-resistant *S. aureus* (VRSA), *Bacillus subtilis* (ATCC 6633), *Staphylococcu aureus* (ATCC 6538P), *Staphylococcus epidermidis* (ATCC 12228), *Escherichia coli* (ATCC 8739), *S. aureus* (ATCC 43300), *P. aeruginosa* (DMH 1), *S. aureus* (DMH 2–DMH 8 and 10–DMH 14), *E. coli* (DMH 9), *Bacillus flexus* and *Proteus aureus*, *Salmonella typhi*, *Klebsiella pneumoniae*, *Bacillus cereus*, *S. aureus* (NCIM-2931), *Pseudomonas aeruginosa* (NCIM-2200), *Proteus vulgaris* (NCIM-2813), *E. coli* (NCIM-2931), *E. coli* (ATCC 8739), and MRSA (ATCC 43300)	[81,86,106,149]
*Tabernaemontana angulata*	Aerial parts and stem	Chloroform, methanol, dichloromethane/methanol, and aqueous	*P. aeruginosa* (ATCC 90270), *S. aureus* (ATCC 6538), and *C. albicans*	[23,28,131]
*Tabernaemontana catharinensis*	Thin branch, leaf root and, stem bark	Alkaloid fraction, ethanol, methanol alkaloids, dichloromethane, n-butanol fractions and 12-methoxy-Nb-methylvoachalotine, Monogagaine and vobparicine	*Mycobacterium tuberculosis* H37Rv, *Mycobacterium avium*, *Mycobacterium kansasii*, *Mycobacterium malmoense*, *Mycobacterium fortuitum*, *Mycobacterium smegmatis*, *Micrococcus* sp., *Enterococcus faecalis*, *Proteus mirabilis*, *S. aureus*, *Aeromonas* sp., *E. coli*, *K. pneumoniae*, *P. aeruginosa*, *B. subtilis* (ATCC 6633), *S. aureus* (ATCC 25923), *S. aureus* MR (ATCC 43300), *S. epidermidis* (ATCC1220228), *E. coli* (ATCC 35218), *Enterobacter cloacae* (ATCC 202), *P. aeruginosa* (ATCC 27853), *Streptococcus faecalis* (ATCC 29232), *K. pneumoniae* (ATCC 700603), *Salmonella enteritidis* (EB 1874/88), *Shigella flexneri* (2EB 7), *Acinetobacter lwoffii* strains, *T. rubrum*, *Enterococcus* sp., and *Citrobacter*	[1,52,88,132,150,151,152,153,154,155]
*Tabernaemontana citrifolia*	Not indicated	Voacangine and ibogaine	*M. tuberculosis*, *M. avium*, and *M. kansasii*	[156]
*Tabernaemontana coronaria*	Leaf	Methanol	*M. tuberculosis* H37Rv (ATCC 2561)	[157]
*Tabernaemontana corymbosa*	Stem bark	Alkaloid fraction	*B. cereus* (ATCC 11778), *P. aeruginosa* (ATCC 27853), *S. aureus* (ATCC 25923), and *E. coli* (ATCC 35218)	[158]
*Tabernaemontana dichotoma*	Root bark, stem bark, stem, bark, leaf, and fruit	Monogagaine, acid aqueous, and ethanol	*B. subtilis*, *B. subtilis* (ATCC 6633), *S. aureus* (ATCC 6538), *P. aeruginosa* (ATCC 9027), and *E. coli* (ATCC 8739)	[11,52]
*Tabernaemontana divaricata*	Leaf, bark, petal, flower, twig, and root	Ethanol, chloroform, petroleum ether, diethyl ether, methanol, aqueous, acetone, alkaloids, 5-oxocoronaridine, alkaloid fraction, Dichloromethane, ethyl acetate, taberdivamines A and B	*K. pneumoniae*, *S. aureus*, *Staphylococcus saprophyticus*, *Streptococcus agalatiae*, *Streptococcus pyogenes*, *E. faecalis*, *S. typhi*, *E. coli*, *Shigella boydii*, *Shigella dysenteriae*, *P. aeruginosa*, *B. cereus*, *Klebsiella* sp., *Streptococcus uberis*, *E. coli* (ATCC 25922), *K. pneumoniae* (ATCC 35657), *Salmonella typhimurium* (MTCC 441), *S. flexneri* (ATCC 29508), *S. aureus* (ATCC25923), *Aeromonas hydrophila*, *S. epidermidis*, *Gardnerella vaginalis*, *Streptococcus agalactiae*, *Propionibacterium acnes*, *Corynebacterium macbinleyi*, *B. subtilis*, *S. faecalis*, *Bacillus megaterium*, *P. mirabilis*, *S. flexneri* (BCH 995), *S. boydii* (8), *Shigella sonnei* (NK 840), *S. dysenteriae* (1), *S. dysenteriae* (9), *Vibrio cholerae* (1023), *V. atherin* (1341), *V. cholerae* (575), *V. cholerae* (1311), *V. cholerae* (756), *E. coli* (RH 07/12), *E. coli* (18/9), *E. coli* (K88), *Enterobacter spp*., *Streptococcus suis*, *Salmonella* sp., *Corynebacterium diphtheriae*(AP596), *S. aureus* (ML 267), *S. aureus* (MTCC 96), *S. aureus* (ATCC 6538), *B. subtilis* (MTCC 441), *Bacillus pumilis* (8241) *P. aeruginosa* (AP585 NLF), *K. pneumoniae* strains, *Salmonella Paratyphi*, *Lacto bacillus*, *P.* vulgaris, and *Enterobacter aerogenes*	[48,54,97,100,136,159,160,161,162,163,164,165,166,167,168]
*Tabernaemontana elegans*	Root	Ethanol, hexane, dichloromethane, and ethyl acetate	*Haemophilus influenzae* (ATCC 49217), *H. influenzae* (NHLS 609), *B. subtilis* (ATCC 6633), *E. faecalis* (ATCC 29212), *S. aureus* (ATCC 1200), *S. aureus* (ATCC 12600), *S. aureus* (NHLS 363), *S. aureus* (NHLS 284), *Streptococcus pneumoniae* (ATCC 49619), *S. pneumoniae* (NHLS 203), *S. pneumoniae* (NHLS 405), *M. tuberculosis* resistant (MRC 3366), *M. tuberculosis* H37RV (ATCC 25177), *M. smegmatis* (ATCC 14468), *E. coli* (ATCC 35218), *K. pneumoniae* (ATCC 13883), and *P. aeruginosa* (ATCC 9027), *M. tuberculosis* H37Rv	[20,140,169]
*Tabernaemontana heterophylla*	Stem	Dichloromethane	*M. tuberculosis*	[170]
*Tabernaemontana pachysiphon*	Leaf, root, and stem bark	Ethanol, Ibogaine, 12 methoxy-ibogamine, 10-methoxyibogamine and 3-R/S-hydroxy-conopharyngine	*S. aureus*, *E. coli*, *B. subtilis*, *Agrobacterium tumefaciens*, *B. subtilis* (ATCC 6633), *S. aureus* (ATCC 6538), *P. aeruginosa* (ATCC 9027), and *E. coli* (ATCC 8739)	[1,142,171]
*Tabernaemontana solanifolia*	Leaf, bark, and root	Voacangine, isovoacangine, coronaridine, heyneanine, isovoacristine, voacangine hydroxyindolenine, vobasine, voachalotine, 12-methoxy-Nb-methylvoachalotine and voacamine	*C. albicans* B311	[69]
*Tabernaemontana stapfiana*	Root and stem	Methanol	*S. aureus*, MDRS, *E. faecalis*, *B. subtilis*, and *S. typhi*	[172]

**Table 7:** Antiamoebic activities of extracts and compounds isolated from *Tabernaemontana* species.

Species	Part/Exudate	Extract	Cell Lines/Models/Methods	References
*Tabernaemontana* *aurantiaca*	Leaf and twig	Ethanol	*Entamoeba* *histolytica*	[155]
*Tabernaemontana* *chippii*	Leaf, root bark, and stem bark	Ethanol	*E. histolytica*	[155]
*Tabernaemontana* *contorta*	Leaf and twig	Ethanol	*E. histolytica*	[155]
*Tabernaemontana* *crassa*	Stem bark	Ethanol	*E. histolytica*	[155]
*Tabernaemontana* *dichotoma*	Leaf	Ethanol	*E. histolytica*	[155]
*Tabernaemontana glandulosa*	Leaf and stem bark	Ethanol	*E. histolytica*	[155]
*Tabernaemontana* *heterophylla*	Leaf and twig	Ethanol	*E. histolytica*	[155]
*Tabernaemontana* *longiflora*	Leaf and twig	Ethanol	*E. histolytica*	[155]
*Tabernaemontana* *orientalis*	Leaf and twig	Ethanol	*E. histolytica*	[155]
*Tabernaemontana* *pachysiphon*	Root bark and stem bark	Ethanol	*E. histolytica*	[155,173]
*Tabernaemontana* *penduliflora*	Stem bark	Ethanol	*E. histolytica*	[165]
*Tabernaemontana* *psorocarpa*	Leaf	Ethanol	*E. histolytica*	[165]
*Tabernaemontana* *undulata*	Stem bark	Ethanol	*E. histolytica*	[165]
*Tabernaemontana* *ventricosa*	Leaf and stem bark	Ethanol	*E. histolytica*	[165]
*Tabernaemontana* species	Root	Not indicated	*E. histolytica*	[174]

**Table 8:** Anticancer activities of extracts and compounds isolated from *Tabernaemontana* species.

Species	Part/Exudate	Extract/Compound	Cell Lines/Models/Methods	References
*Tabernaemontana angulata*	Stem	Chloroform and methanol	Human breast adenocarcinoma (MCF-7), human prostate cancer (PC-3), human lung cancer (NCI-H460), human colon cancer (KM-12), human glioblastoma (SF-268), and myeloma (RPMI-8226)	[23]
*Tabernaemontana alternifolia*	Stem bark and leaf	Aqueous, hexane, ethyl acetate, and methanol	Vero cell line, human gastric carcinoma (A-549, MCF-7, AGS), human colon adenocarcinoma (COLO 320 DM and Vero cell lines)	[84,149]
*T* *abernaemontana bovina*	Aerial parts	Taberdivarine C–F	Human cervical carcinoma (HeLa), human breast adenocarcinoma (MCF-7), and colon carcinoma (SW480)	[199]
*Tabernaemontana bufalina*	Aerial parts	3′-(2-oxopropyl)-19,20 dihydro-tabernamine, 3′-(2 oxopropyl) ervahanine B, 19,20-dihydrovobparicine, ibogamine, coronaridine, voacangine, hainanervatasine, 3-(2-oxopropyl) coronaridine, 3,3′-(oxopropyl) dicoronaridine, isotabernamine, taberdivarines C, taberdivarine D, tabernaelegantine B, tabernaelegantinine B, 19,20-dihydrotabernamine A, taberdivarine E, ervadivaricatine B, taberdivarine F tabernaecorymbosine A, tabernaelegantine C, tabernaelegantine A, and 19-(2-oxopropyl)-conoduine	Small cell lung carcinoma (A-549), human breast adenocarcinoma (MCF-7)	[200]
*T* *abernaemontana calcarea*	Whole plant	Voacristine, isovoacristine, 19-epi-voacristine, hydroxytabernanthine,11-hydroxycoronaridine, 19-epi-heyneanine, 3(R/S)-19-epi-3-oxovoacristine, hydroxyindolenine, 10-methoxyibogamine (ibogaine), and 11-methoxyibogamine	Ovarian cancer (A2780)	[201]
*Tabernaemontana catharinensis*	Leaf, seed, root, bark, and stem	Coronaridine, tabersonine, olivacine, coronaridine-hydroxyindolenine, catharinensine, decarbomethoxyvoacamine and tabernamine, aqueous, ethanol, and alkaloid fraction	Ehrlich’s carcinoma, sarcoma 180, human breast carcinoma (SK-BR-3), melanoma tumor cells (SK-BR-3 and C-8161), laryngeal carcinoma (Hep-2 and normal 3 T3 cell lines), human melanoma cell lines (A375, WM1366, SK-MEL-28)and a normal skin cell line (CCD-1059Sk), adenocarcinomic human alveolar basal epithelial cells (A549), Vero cells (African green monkey kidney epithelial cells)	[33,69,118,189,202,203,204,205]
*Tabernaemontana contorta*	Root	Contortarine A, 16-epi-pleiomutinine and N4-chloromethyl-pleiomutinine	Quinone reductase induction (QR) and NF-kB inhibition assay	[206]
*Tabernaemontana corymbosa*	Stem bark, leaf, and twig	Alkaloid fraction, jerantinine A, jerantinine C, jerantinine B, jerantinine B acetate, jerantinine D, jerantinine E, jerantinine F, conodiparine A, oxoconodiparine A, conodiparine B, oxoconodiparineB, vobasidine A, vobasidine B, vobasidine C, vobasidine D, taipinisinetabernaemontanine, dreagamine, vobasine, 16-epivobasine, vobasenal, 16-epivobasenal and vincristine, 16-decarbomethoxy-voacaminepseudoindoxyl, conolutinine, lirofoline A, conoliferine, conomicidine A, conomicidineB, ibogamine, ibogaine, ibogaine-7-hydroxyindolenine, iboxygaine, iboluteine, coronaridine, heyneanine, 19-epi-heyneanine, 7(R)-geissoschizol oxindole, 7(S)-geissoschizol oxindole, 16(R),7(R)-19,20-eisositsirikine oxindole, affinisine, voachalotine, norfluorocurarine, antirhine, velbanamine, 20(S) hydroxy-1,2 dehydro-pseudo-aspi-dospermidine, tabercorines A–C, acetyl-tabernaecorymbosine A, tabernaricatine A, tabernaricatine B, tabernaricatine D, 16-decarbomethoxyvoacamine, conodurine and tabernaecorymbosine, taburnaemines A–I, tabernaecorymbosine A, tabernaricatineC, tabernaricatine D, 16′-decarbo-methoxytabernaecorymbosine, tabercorymines, and conofolidine	Human non-small lung carcinoma (A549), human cervical carcinoma (C33A), human oral epidermoid carcinoma (vincristine resistant KB, vincristine-sensitive (KB/S), and vincristine-resistant cells KB/VJ300), human colon colorectal (HCT-116 cells, HT-29), human breast adenocarcinoma (MDA-MB-231, MCF-7), human breast cancer (MDA-468), human hepatoma (Hep-G2 and SMMC-7721) cells, human leukemia (HL-60), hepatocarcinoma cell line (SMMC-7721), lung and colon carcinoma (SW480), human carcinoma (KB, and P-glycoprotein over-expressing multi-drug resistant KB cells) and human carcinoma (KB, KB-VIN cells)	[158,207,208,209,210,211,212,213,214,215,216,217,218]
*Tabernaemontana divaricata*	Leaf, root, stem, flower, stem bark aerial parts and whole plant	Chloroform, petroleum ether, methanol, ethylacetate, hexane, ethanol, hydroalcohol, acetone, aqueous, dichloromethane, chloroform-5-oxocoronaridine, 3-oxocoronaridine, coronaridine, ibogamine, tabernaemontamine, vobasine, voacamine, tabernaricatines A–F, bisindole alkaloids, 16-decarbo-methoxyvoacamine, tabernaecorymbosine A, isovoacangine, heyneanine, voacangine, 19acetonyliso-voacangine, vincadiffine, difforlemeine, voaphylline, hecubine, voacangine hydroxyindolenine, voacryptine, 19S-heyneanine, 19S-voacangarine, lo-methoxyeglandine-noxidelo-hydroxycoronaridine, heyneatine, O-acetyl vallesamine, pericalline, dihydrocondylocarpine, camptothecin, 9-methoxycamptothecin, conoduramine, and coronaridinehydroxyindolenine	Human colon carcinoma (HCT-15, HT-29, and 502713), human breast adenocarcinoma (MCF-7), human prostate cancer (PC-3), leukemia (HP-1), brine shrimp lethality, lung carcinoma large cell (COR-L23), laryngeal carcinoma (Hep-2), Vero cells, sarcoma 180, cloned Chinese hamster lung fibroblast (V_79_ cells), human colon cancer (HT-29), human small cell lung carcinoma (A-549), human hepatic cancer (HepG-2), human and rat normal skeletal muscle cell cultures (L6), renal cell carcinoma in Wistar rats, human myeloid leukemia (HL-60), hepatocellular carcinoma (SMMC-7721), colon cancer (SW480), human breast cancer (BC1), human oral epidermoid carcinoma (KB), and KB drug-resistant strain (KB-V1), human prostate cancer (LNCaP), human lung cancer (Lu1), murine, lymphocytic leukemia (P388), human glioma (U373), human epidermoid carcinoma (A431), human colon cancer (Col2), human fibrosarcoma (HT), human melanoma (Mel2), hormone-dependent breast cancer (ZR-75-1), Kl3-V1 cells, and human leukemia (MOLT4)	[54,96,160,193,219,220,221,222,223,224,225,226,227,228,229]
*Tabernaemontana elegans*	Whole plant and root	Ethanol, ethyl acetate, indole alkaloids, tabernaelegantinine B, tabernaelegantine C, dregamine, eleganine A, tabernine A–C, 16-epi-dregamine, and monomeric indole bisindole alkaloids	Human macrophages (THP-1 cells), cell viability screening (trypan blue dye exclusion assay), human hepatoma (HuH-7, HCT-116, and SW620 cells), human cervical epithelial carcinoma (HeLa), African green monkey kidney cells (Vero), and human embryonic kidney (HEK-293),	[169,230,231,232]
*Tabernaemontana hystrix*	Root bark	Voacamine	Lymphoblastoid (CEM-WT), osteosarcoma (U-2 OS-WT), and drug-resistant cell lines (CEM-R and U-2 OS-R)	[233]
*Tabernaemontana laeta*	Stem bark	Ethyl acetate	Human carcinoma (KB cells)	[64]
*Tabernaemontana pandacaqui*	Leaf	Ethanol	Human primary renal cell carcinoma (786-0), colon adenocarcinoma grade II (HT-29), ovarian carcinoma (OVCAR-3), melanoma (SK-MeI-28), glioblastoma (SNB-19), human breast adenocarcinoma (MCF-7), human prostate cancer (PC-3), and small cell lung carcinoma (A549)	[234]
*Tabernaemontana solanifolia*,	Leaf, bark, and root	12-methoxy-Nb-methyl-voachalotine	Leukemia (3PS31)	[69]
*Tabernaemontana salzmannii*,	Root bark	Voacangine	Monocytic leukemia (THP-1)	[67]

**Table 9:** Antiacetylcholinesterase activities of extracts and compounds isolated from *Tabernaemontana* species.

Species	Plant Organ/Exudate	Extract/Compound	Methods	References
*T* *abernaemontana amygdalifolia*	Latex	Ethanol and methanol	Ellman’s method	[246]
*T* *abernaemontana australis*	Stem and stalk	Ethanol	Ellman’s method	[242]
*Tabernaemontana divaricata*	Leaf, flower, root, stem, and latex	Methanol, ethanol, phosphate buffer, petroleum ether, dihydrotabernamine, 19,20-dihydro-ervahanine A, conodurine, tabernaelegantine A, Taberhanine, voafinine, *N*-methylvoafinine, voafinidine, voalenine, conophyllinine, conophylline, and conofoline,	Ellman’s method	[51,53,193,237,238,241,247,248]
*T* *abernaemontana iboga*	Root bark	Ibogaine and physostigmine	Ellman’s method	[249]
*Tabernaemontana* *laeta*	Stalk and stem	Ethanol	Ellman’s method	[61]
*Tabernaemontana pandacaqui*	Flower	Methanol	Ellman’s method	[245]

**Table 10:** Biological activity of synthesized silver nanoparticles using extracts isolated from *Tabernaemontana* species.

Species	Plant Organ/Exudate	Extract	Biological Activity	References
*Tabernaemontana divaricata*	Leaf	Aqueous and ethanol	Cytotoxicity (human breast cancer cell line-MCF-7), In vitro antioxidant, anticataractogenic, and in vivo anticataractogenic	[101,256,267]

## Data Availability

Not applicable.
